# A Study to Determine the Frequency Rate of Scoliosis Disorder and Compare the Anthropometric Characteristics of Normal versus the Scoliosis Diagnosed Students

**DOI:** 10.5539/gjhs.v8n9p288

**Published:** 2015-01-31

**Authors:** Gholamreza Khosravi, Mohammad Reza Sharif, Erfan Khosravi, Fatemeh Kardan, Hamed Haddad Kashani, Mansour Sayyah

**Affiliations:** 1 Trauma Research Center, Kashan University of Medical Sciences, Kashan, Iran; 2 Pediatric Department, Kashan University of Medical Sciences, Kashan, Iran; 3 Faculty of Medicine, Kashan University of Medical Sciences, Kashan, Iran; 4 Department of Physical Sport, University of Kashan, Kashan, Iran; 5 Anatomical Sciences Research Center, Kashan University of Medical Sciences, Kashan, Iran

**Keywords:** deformity, scoliosis, student, elementary school

## Abstract

**Introduction:**

Postural deformities are commonly acquired disorders that occur throughout the life. The purpose of this research was to determine and compare the frequency of scoliosis disorder and anthropometric characteristics of normal versus the disordered Students.

**Materials and Methods:**

This was a cross-sectional study that was performed on 1416 girls and boys of elementary school students in the city of Kashan in education year 2010-2011. Adams bending test was employed to examine 1416 students to identify the disorder. Seca scale was employed to measure weight and inflexible tape was used to measure the height of students. SPSS software was employed to analyze the data.

**Results:**

The result of analysis showed that 63.8 percent of students were boys and 36.2 percent were girls. The frequency of scoliosis in boys and girls was 29.8 and 24.2 percent, respectively. Independent t-test result showed that there was a significant difference between the height and weight of normal versus the scoliosis identified boys and girls student (P=0.004, 0.031; 0.0001, 0.041).

**Conclusion:**

These types of studies are conducted regularly to identify poor postural cases at an early stage. The identification of acquired deformities at an early stage is important since it provides the opportunity to take the appropriate measures to correct them. Early identification of scoliosis is vital to maximize effectiveness of treatment.

## 1. Introduction

Human posture undergoes considerable changes during the life span. While most of the changes are desirable and are considered as development, there are occasions when the change is faulty and may cause limitation to normal functioning. Faulty posture is common in children and teens with a wide spectrum of symptoms and causes. There are numerous researches conducted to examine the common deformities that may occur due to various reasons (Zhang et al., 2015; Smyrnis et al., 2015; Penha et al., 2005; Nery et al., 2010; Sharma et al., 2013; Drerup et al., 1996; Hierholzer et al., 2002; Mahlknecht 2007; Płaszewski et al., 2014; Eivazi et al., 2012; Konieczny et al., 2013). Certainly, postural deformities that have no inherited roots are acquired. The time of acquisition or affliction varies depending on the condition when the person starts to experience the initiation of deformity. Since the demonstration of deformity is not noticeable at the early stages and only expert eyes may become aware of such incidence, individuals at risk hardly if ever realize that they are facing postural deformities. For this reason, many researches have been conducted to examine the prevalence of postural deformities. The majority of the researches have focused on the prevalence of the problem in young population including children and particularly school children (Latalski et al., 2013; Weinstein et al., 1981; Nery et al., 2010). The prevalence of deformities includes spinal deviations from its natural shape and most researches are designed to examine this type of abnormality (Trobisch et al., 2010; Bunnell et al., 2005; Adams 1865; Stagnara et al., 1982). Since the incidence and prevalence of postural deformity depends on many factors such as environmental and cultural habits of every population, it is necessary to contact this type of research in different environment and populations. Therefore, this research was designed to examine the prevalence of scoliosis and its relationship with some anthropometric indices including height and weight of boys’ and girls’ students in elementary schools.

## 2. Methods and Materials

This was a cross-sectional study that was performed on 1416 girls and boys of elementary school students in the city of Kashan in education year 2010–2011. Adams bending test was employed to examine 1416 students to identify the disorder. The procedures were explained to the participants and they voluntarily participated in the protocol. All the measurements were made in the gymnasium during the physical education programs. Seca scale was employed to measure weight and inflexible tape was used to measure the height of students. SPSS software was employed to analyze the data.

## 3. Results

The result of analysis showed that 63.8 percent of students were boys and 36.2 percent were girls (Table 1). The frequency of scoliosis in boys and girls was29.8 and 24.2 percent, respectively. Independent t-test was employed to compare the height, weight, body mass index (BMI and waist to hip ratio (W/H) of boys and girls students in regard to the presence or absence of scoliosis (Table 3). The result of analysis indicated that there was a significant difference between the height and weight of normal boys and girls and boys and girls who were identified as scoliosis condition based on Adams test (P=0.004, 0.031; 0.0001, 0.041). The boys with normal posture were significantly heavier and taller that their counterpart in the scoliosis condition group. However, the normal girl had significantly lighter weight than the scoliosis group.

**Table 1:** Frequency distribution of elementary students according to the sex

Sex	Frequency	Valid Percent	Cumulative Percent
Boy	904	63.8	63.8
Girl	512	36.2	100.0
Total	1416	100.0	

**Table 2:** Frequency distribution of scoliosis according to gender

Sex	Condition	Frequency	Percent	Cumulative Percent
Boy	normal	635	70.2	70.2
	abnormal	269	29.8	100.0
	Total	904	100.0	
Girl	normal	388	75.8	75.8
	abnormal	124	24.2	100.0
	Total	512	100.0	

**Table 3:** Characteristics of elementary students according to posture

Sex	Variables	Conditions			

		normal		scoliosis	
		mean	S.D.	mean	S.D.
Boy	Height(cm)	143.32	9.2	141.40	8.8
	Weight (kg)	38.06	11.03	36.36	10.35
	BMI(kgm^2^)	18.25	3.68	17.91	3.46
	W/hip	0.88	.065	0.89	.058

Girl	Height(cm)	145.60	7.73	149.10	6.64
	Weight (kg)	41.10	9.53	43.10	7.56
	BMI(kgm^2^)	19.28	3.46	19.30	2.62
	W/hip	0.82	0.064	0.81	0.059

In addition, there was a significant difference between the waist to hip ratio of the boys (P=0.022) but not the girls (P=0.175). No significant differences was fount between the BMI of normal and scoliosis identified boys and girls (P>0.05). These results are presented in Table 4.

**Table 4:** Comparing the characteristics of elementary students according to posture

Sex	Variables	t	Sig. (2-tailed)
Boy	Height(cm)	2.904	.004*
	Weight (kg)	2.165	.031*
	BMI(kgm2)	1.307	.192
	W/hip	-2.301	.022*
Girl	Height(cm)	-4.491	.000*
	Weight (kg)	-2.063	.040
	BMI(kgm2)	-.047	.963
	W/hip	1.360	.175

* *Significant at alpha level= 0.05*.

## 4. Discussion

Test for identifying scoliosis has been employed for more than half a century. These tests are proven to be useful in estimating the prevalence of scoliosis in one hand and valid for providing the opportunity to prevent further deterioration of the condition in those who are afflicted to the disorder in other hand. Early detection of the condition may help the authorities in charge of health to introduce no operative treatment, such as wearing an orthosis that has been shown to be effective by numerous outcome studies. Adams was one of the pioneers in this subject that introduced a simple procedure that indirectly identify spinal deformity simply by bending forward and observing the deformity (Stagnara et al., 1982; Zurita et al., 2008). Stagnara claimed that the spine has a fixed rotational direction (Stagnara et al., 1982). Posterior elements attempt to rotate towards the concave side, and they try to make the shortest advancement just like an athlete running at the extreme interior lane of marathon course. Therefore, the perpendicular distance is shorter on the posterior side of the vertebrae compared to the relevant distance on the anterior side (Zurita et al., 2008). The use of instrument to assess scoliosis has been the subject of some studies. For instance, Côté and associates (1998) compared the Scoliometer with Adam’s forward bend tests and reported thatAdams’ test had adequate interexaminer reliability for the assessment of thoracic curves (Côté et al., 1998). Further, they concluded that the Scoliometer has a high level of inter examiner measurement error that limits its use as an outcome instrument. Because Adam’s forward bend test is more sensitive than the Scoliometer, the authors believe that it remains the best noninvasive clinical test to evaluate scoliosis. Thus, the results of this study were sufficiently reliable to compare them with the result of other researchers. The prevalence of scoliosis was 29.8 percent in boys compared to 24.2 percent in girls. The higher prevalence of scoliosis in boys compared to girls has been reported by other researches. Scientists conducted a research in Turkey and reported that the prevalence of scoliosis was 47 percent; a much higher rate than the rate observed in the present research (Cilli et al., 2009).

In regard to the relationship between the gender and scoliosis disorder, contradictory findings have been reported. While several studies (Suh et al., 2011; Daruwalla et al., 1985) have reported a higher Cobb angels in girls than in boys claiming that scoliosis in girls progresses to a higher grade of severity. For patients with a Cobb angle of more than 30° the prevalence ratio gets as high as 10:1 (Soucacos et al., 1997; Weinstein et al., 2008; Raggio, 2006; Luk et al., 2010; Ueno Ueno et al., 2011). The prevalence of severe scoliosis is much higher for girls than for boys; however, Weijun (2012) reports a higher prevalence of atypical curve types in boys with Cobb angles of more than 20° than in girls, and a higher risk of progression in the main thoracic right convex curve. Kratenová and associates (2007) conducted a research in Czeck and found that the prevalence of poor posture was significantly higher in boys than girls (Kratenová et al., 2007). The result of this was in agreement with the findings of Weijun (2012) and Kratenová and associates (2007) who showed that boys suffered more than girls from scoliosis disorder. These discrepancies may be attributed to age of subjects or other characteristics of the subjects that were not included in the research design. In the present research, the heavier boys were more likely to suffer from scoliosis; the girls on average with less weight were more likely to face scoliosis disorder. The same contradictory findings were observed for the height in boys and girls. While the taller girls were more likely to face scoliosis, the condition was the opposite for the boys.

## 5. Conclusion

Despite the fact that different tools or methods are used to examine the frequency or prevalence of, it is evident that this type of postural deformities is present in all parts of the world. This type of studies is necessary to identify cases and take appropriate measure to correct the problem. The early identification of the acquired deformities makes the treatment or correction much easier than attempting to correct them at the later of life. Early identification of scoliosis is vital to maximize effective treatment, support the child and family, and optimize holistic health. This study was limited in scope since the elementary students were examined. More age groups with more variables in addition to employing tests with higher sensitivity such as x-ray examination are needed to provide more comprehensive information about the development of poor or faulty postures.
